# Insulin resistance contributes to racial disparities in breast cancer prognosis in US women

**DOI:** 10.1186/s13058-020-01281-y

**Published:** 2020-05-12

**Authors:** Emily J. Gallagher, Kezhen Fei, Sheldon M. Feldman, Elisa Port, Neil B. Friedman, Susan K. Boolbol, Brigid Killelea, Melissa Pilewskie, Lydia Choi, Tari King, Anupma Nayak, Rebeca Franco, Daliz Cruz, Irini M. Antoniou, Derek LeRoith, Nina A. Bickell

**Affiliations:** 1 Division of Endocrinology, Diabetes and Bone Disease, Icahn School of Medicine at Mount Sinai, 1428 Madison Avenue, Box 1055, New York, NY 10029 USA; 2 Department of Medicine, Icahn School of Medicine at Mount Sinai, New York, NY USA; 3 Tisch Cancer Institute at Mount Sinai, Icahn School of Medicine at Mount Sinai, New York, NY USA; 4 Department of Population Health Science and Policy, Center for Health Equity & Community Engaged Research, Icahn School of Medicine at Mount Sinai, New York, NY 10029 USA; 5 Center for Health Equity & Community Engaged Research, Icahn School of Medicine at Mount Sinai, New York, USA; 6 Department of Surgery, Columbia University Medical Center, New York, NY USA; 7 Department of Surgery, Icahn School of Medicine at Mount Sinai, New York, NY USA; 8 Department of Surgery, Mercy Medical Center, Baltimore, MD USA; 9 Department of Surgery, Mount Sinai Beth Israel, New York, NY USA; 10 Department of Surgery, Yale School of Medicine, New Haven, CT USA; 11 Department of Surgery, Memorial Sloan Kettering Cancer Center, New York, NY USA; 12 Department of Surgery, Wayne State University School of Medicine, Detroit, MI USA; 13 Department of Pathology and Laboratory Medicine, Hospital of the University of Pennsylvania, Philadelphia, PA USA

**Keywords:** Breast cancer, Cross-sectional study, Prognosis, Disparities, Insulin resistance, Insulin receptor, Insulin-like growth factor receptor

## Abstract

**Background:**

Racial disparities in breast cancer survival between Black and White women persist across all stages of breast cancer. The metabolic syndrome (MetS) of insulin resistance disproportionately affects more Black than White women. It has not been discerned if insulin resistance mediates the link between race and poor prognosis in breast cancer. We aimed to determine whether insulin resistance mediates in part the association between race and breast cancer prognosis, and if insulin receptor (IR) and insulin-like growth factor receptor (IGF-1R) expression differs between tumors from Black and White women.

**Methods:**

We conducted a cross-sectional, multi-center study across ten hospitals. Self-identified Black women and White women with newly diagnosed invasive breast cancer were recruited. The primary outcome was to determine if insulin resistance, which was calculated using the homeostatic model assessment of insulin resistance (HOMA-IR), mediated the effect of race on prognosis using the multivariate linear mediation model. Demographic data, anthropometric measurements, and fasting blood were collected. Poor prognosis was defined as a Nottingham Prognostic Index (NPI) > 4.4. Breast cancer pathology specimens were evaluated for IR and IGF-1R expression by immunohistochemistry (IHC).

**Results:**

Five hundred fifteen women were recruited (83% White, 17% Black). The MetS was more prevalent in Black women than in White women (40% vs 20%, *p* < 0.0001). HOMA-IR was higher in Black women than in White women (1.9 ± 1.2 vs 1.3 ± 1.4, *p* = 0.0005). Poor breast cancer prognosis was more prevalent in Black women than in White women (28% vs 15%. *p* = 0.004). HOMA-IR was positively associated with NPI score (*r* = 0.1, *p* = 0.02). The mediation model, adjusted for age, revealed that HOMA-IR significantly mediated the association between Black race and poor prognosis (*β* = 0.04, 95% CI 0.005–0.009, *p* = 0.002). IR expression was higher in tumors from Black women than in those from White women (79% vs 52%, *p* = 0.004), and greater IR/IGF-1R ratio was also associated with higher NPI score (IR/IGF-1R >  1: 4.2 ± 0.8 vs IR/IGF-1R = 1: 3.9 ± 0.8 vs IR/IGF-1R < 1: 3.5 ± 1.0, *p* < 0.0001).

**Conclusions:**

In this multi-center, cross-sectional study of US women with newly diagnosed invasive breast cancer, insulin resistance is one factor mediating part of the association between race and poor prognosis in breast cancer.

## Introduction

The metabolic syndrome is a set of biological factors (abdominal obesity, hypertension, dyslipidemia, and dysglycemia), associated with an increased risk of a number of diseases including cancer [1, 2]. In breast cancer, the metabolic syndrome has been associated with an increased risk of developing cancer, and a worse prognosis [3, 4]. In the 2007–2012 US National Health and Nutrition Examination Survey (NHANES), the overall prevalence of the metabolic syndrome was 34% [5]. Non-Hispanic Black women were found to be 20% more likely to have the metabolic syndrome than non-Hispanic White women [5].

Non-Hispanic Black women still experience 39% higher rates of breast cancer mortality than non-Hispanic White women, despite similar incidence [6]. Black women have higher rates of triple-negative breast cancer (TNBC) [7–10] and also experience higher mortality from estrogen receptor (ER)-positive breast cancers [11]. A number of complex factors have been proposed to contribute to the disparities in breast cancer mortality including access to care, screening, and treatment; socioeconomic factors; systemic metabolic conditions; tumor biology; and epigenetic and genetic factors [12, 13].

Insulin resistance and endogenous hyperinsulinemia are key features that underlie the development of the metabolic syndrome [14]. Studies have reported that women with early-stage breast cancer and endogenous hyperinsulinemia have decreased rates of recurrence-free survival [9]. Preclinical studies have found that hyperinsulinemia promotes the growth and metastasis of breast cancers by activating the insulin receptor (IR)/insulin-like growth factor 1 receptor (IGF-1R) signaling pathways [15, 16]. Differences in hepatic insulin metabolism have been reported between African American and European American women, leading to higher circulating insulin levels in African American women [17]. Whether hyperinsulinemia and tumor IR expression contribute to the racial disparities in breast cancer prognosis has not previously been explored.

In this study, we aimed to determine whether insulin resistance (determined by the homeostatic model assessment of insulin resistance, HOMA-IR) mediates the association between race and breast cancer prognosis, which was determined by the Nottingham Prognostic Index (NPI). We additionally explored whether expression of the IR and IGF-1R in the breast cancer cells was associated with race and worse breast cancer prognosis.

## Methods

### Patient accrual, data collection, and laboratory measurements

In this cross-sectional study, women were recruited shortly after the diagnosis of a new primary invasive breast cancer from ten US hospital sites, including five academic medical centers and five community hospitals in five states: New York, New Jersey, Connecticut, Maryland, and Michigan. A survey assessing breast cancer and metabolic syndrome risk factors, anthropometric measures, fasting blood, and tissue samples was collected. Institutional Review Board (IRB) approval was obtained from all participating sites. Recruitment began in March 2013. Women who were eligible for the study were aged 21 years or more and self-identified as being Black women (including Hispanic Black women) or White women (excluding Hispanic White women). Exclusion criteria included women with type 1 or type 2 diabetes being treated with oral or injectable medication; previous bariatric surgery; glucocorticoid treatment within 2 weeks of recruitment for blood testing, biopsy, or surgical resection; end-stage renal disease or hepatic cirrhosis; prior organ transplantation; and receipt of neoadjuvant chemo- or hormonal therapy for breast cancer prior to blood tests or tissue sampling. We excluded women with type 2 diabetes on oral or injectable medications in order to evaluate HOMA-IR in the absence of medication that could affect insulin sensitivity, secretion, or measurement of endogenous insulin levels; however, it led to a lower rate of eligible Black women who had higher rates of type 2 diabetes.

Clinical data recorded included self-reported smoking, alcohol intake, diet, physical activity, education, income, and health insurance. Breast cancer screening history was also recorded and was defined as inadequate if women between the ages of 50–74 years had not had a mammogram in the 2 years prior to the mammogram that led to the current diagnosis of breast cancer. Charlson Comorbidity Index was calculated [18].

At the study visit, each participant had height (m) and weight (kg) measurements recorded from which body mass index (BMI) was calculated (kg/m^2^). Waist circumference (cm) was measured using the NHANES procedures [19]. Blood pressure was obtained using a clinical electronic blood pressure monitor. Venous blood was drawn after an overnight fast (minimum 8 h) for plasma glucose, serum insulin, C-peptide, and a lipid panel [total cholesterol, high-density lipoprotein (HDL) cholesterol, low-density lipoprotein (LDL) cholesterol, and triglycerides (TG)]. Insulin resistance was calculated by the HOMA-IR equation: [fasting glucose (mg/dL) × fasting serum insulin (μU/mL)]/405.

### Definitions of obesity, metabolic syndrome, and insulin resistance

Obesity was defined as a BMI of ≥ 30 kg/m^2^, or by the Adult Treatment Panel (ATP) III waist circumference (WC) cutoff of ≥ 88 cm. The metabolic syndrome was defined as having three or more of the following five criteria: (1) WC ≥ 88 cm; (2) triglycerides ≥ 150 mg/dL, or on treatment for hypertriglyceridemia; (3) HDL < 50 mg/dL; (4) fasting glucose ≥ 100 mg/dL; (5) systolic blood pressure ≥ 130 mmHg or diastolic blood pressure ≥ 85 mmHg, or on treatment for hypertension [20]. Insulin resistance was defined as a HOMA-IR score of > 2.8, the upper quartile of the US population, reported by NHANES III [21].

### Breast cancer subtype, stage, and prognosis determination

Clinical pathology reports were obtained from the patients’ electronic medical records to classify breast cancers as ER positive, HER2 overexpressing, or TNBC. The Nottingham Prognostic Index (NPI) score was calculated as 0.2 × tumor size (cm) + lymph node (LN) stage (1: LN negative, 2: 1–3 positive LNs, 3: ≥ 4 positive LNs) + histological grade (1, well-differentiated; 2, moderately differentiated; 3, poorly differentiated) [22]. Improved NPI (iNPI) was defined as previously described, adding one point for HER2 positivity, and subtracting one point for progesterone receptor (PR) positivity [23]. Tumor grade was defined by the Nottingham combined histological grade (NCHG), as recommended by the American Joint Committee on Cancer (AJCC) criteria [24]. Poor prognosis was defined as an NPI score of > 4.4, or an iNPI > 5.4 [25, 26].

### Immunohistochemistry staining and analysis

The IR and IGF-1R expression was evaluated by immunohistochemistry (IHC) in compliance with the REMARK guidelines [27]. IHC methods with antibody sources and concentrations are detailed in Supplementary file 1. The Allred scoring system was used to assess the intensity of cell staining, and the proportion of tumor stained positive for IR and IGF-1R [28], as previously described [29, 30]. As no standard cutoffs have been determined for IR and IGF-1R staining, 0–4 was considered “low” and > 5 was considered “high.” For the IR/IGF-1R ratio, we assigned a score of > 1 if the level of IR expression was greater than IGF-1R, 1 if IR was equal to IGF-1R expression, and < 1 if IGF-1R was greater than IR expression.

### Statistical analysis

Basic statistics were used to describe patient characteristics. Means and standard deviations were presented for continuous variables, and frequencies and proportions were presented for categorical variables. Group comparisons used *t* tests on continuous, and *χ*^2^ tests on categorical variables. The sample size was determined based on estimated rates of insulin resistance in White women (20%) and Black women (33%) and a main effect OR >  1.5 between insulin resistance and poor prognosis breast cancer between Black women and White women. We used Andrew Hay’s INDIRECT macro to estimate the path coefficients in an adjusted mediator model and used bootstrapping to estimate confidence intervals for indirect effects of race on prognosis through HOMA-IR. It allowed for adjustment for the potential influence of covariates not proposed as being mediators in the model. SAS 9.4 software (SAS Institute, Cary, NC) was used for all statistical analyses. All tests were two-sided and statistical significance was set at 0.05 level.

## Results

### Patient characteristics

Five hundred fifteen women with newly diagnosed breast cancer were consented for inclusion in the study. Patient characteristics are shown in Table 1. Eighty-three percent (*n* = 428) self-identified as non-Hispanic White women, and 17% (*n* = 87) self-identified as Black women. There was no difference in age at breast cancer diagnosis between Black and White women. More White women (48%) than Black women (36%) were current smokers (*p* = 0.04). Additionally, more White women (29%) consumed more than 2 alcoholic drinks per week than Black women (5%), *p* < 0.0001. There was no difference in the percent of Black women and White women with commercial health insurance. More Black women (75%) than White women (31%) had an annual income of <$75,000 (*p* < 0.0001) and had less than a college education (*p* < 0.0001). Sixty-eight percent of Black women had a Charlson Comorbidity Index of 1 or more, compared with 53% of White women (*p* = 0.01).

**Table 1:** Patient and tumor characteristics by self-identified race

	Total	White	Black	*p* value
***N*(%)**	515 (100%)	428 (83%)	87 (17%)	
Age, mean (SD)	58.3 (12.4)	58.3 (12.3)	58.1 (13.2)	0.9
**Metabolic characteristics**				
BMI, mean (kg/m^2^)	27.1 (6.4)	26.3 (5.9)	31.2 (6.8)	< .0001
Obese (BMI ≥ 30 kg/m^2^)	120 (24%)	79 (19%)	41 (47%)	< .0001
Abdominal obesity (WC > 88 cm)	332 (72%)	250 (67%)	82 (96%)	< .0001
Metabolic syndrome	120 (23%)	85 (20%)	35 (40%)	< .0001
**Comorbidities**				
Charlson Comorbidity Index (≥ 1)	284 (55%)	225 (53%)	59 (68%)	0.01
**Tumor characteristics**				
Tumor size, cm	1.5 (1.2)	1.5 (1.2)	1.7 (1.2)	0.2
Stage				0.05
I	327 (64%)	281 (66%)	46 (53%)	
II	169 (33%)	133 (31%)	36 (41%)	
III	19 (4%)	14 (3%)	5 (6%)	
Insulin resistant (HOMA-IR > 2.8)	57 (11%)	42 (10%)	15 (17%)	0.04
NPI > 4.4	87 (17%)	63 (15%)	24 (28%)	0.004
iNPI >5.4	17 (3%)	9 (2%)	8 (9%)	0.001
**Tumor hormone receptor status**				
Estrogen receptor positive	433 (88%)	371 (91%)	62 (77%)	0.0003
Progesterone receptor positive	405 (83%)	346 (85%)	59 (73%)	0.01
Her2 positive	31 (6%)	25 (6%)	6 (8%)	0.65
Triple negative	38 (8%)	28 (6%)	13 (16%)	0.01
**Screening behavior**				
Mammogram ≥ 2 years before diagnosis	108 (22%)	91 (22%)	17 (20%)	0.6
**Lifestyle factors**				
Smoking: never smoker	265 (54%)	211 (52%)	54 (64%)	0.04
Alcohol: > 2 drinks/week	122 (25%)	118 (29%)	4 (5%)	< .0001
Diet: very good/excellent diet	264 (54%)	239 (59%)	25 (30%)	< .0001
Physical activity: sedentary	236 (49%)	184 (47%)	52 (60%)	0.02
**Socioeconomic factors**				
Education: < college education	85 (18%)	54 (14%)	31 (38%)	< .0001
Income: < $75,000/year	70 (40%)	43 (31%)	27 (75%)	< .0001
Insurance: commercial insurance	436 (89%)	364 (90%)	72 (84%)	0.1

Obesity was more prevalent in Black women than in White women, whether defined by BMI (47% vs 19%, *p* < 0.0001) or as waist circumference (96% vs 67%, *p* < 0.0001). The metabolic syndrome was also more prevalent in Black women than in White women (40% vs 20%, *p* < 0.0001). HOMA-IR was higher in Black women (1.9 ± 1.2) than in White women (1.3 ± 1.4), *p* = 0.0005. Seventeen percent of Black women had HOMA-IR > 2.8, compared with 9% of White women (*p* = 0.03). More White women than Black women reported that their diet was “very good/excellent” (59% vs 30%, *p* < 0.0001), and engaged in moderate or more physical activity (53% vs 40%, *p* = 0.02).

### Breast cancer screening and tumor characteristics

There was no difference in the rate of breast cancer screening or the AJCC stage at diagnosis between Black women and White women. However, 28% of Black women had an NPI of > 4.4, compared with 15% of White women (*p* = 0.004). Rates of ER-positive breast cancer were higher in White women than in Black women (91% vs 77%, *p* = 0.0003). There were no differences in the percent of women with HER2-positive breast cancer. TNBC was more common in Black women than in White women (16% vs 6%, *p* = 0.01).

### Association between insulin resistance and breast cancer prognosis

**Fig. 1:**
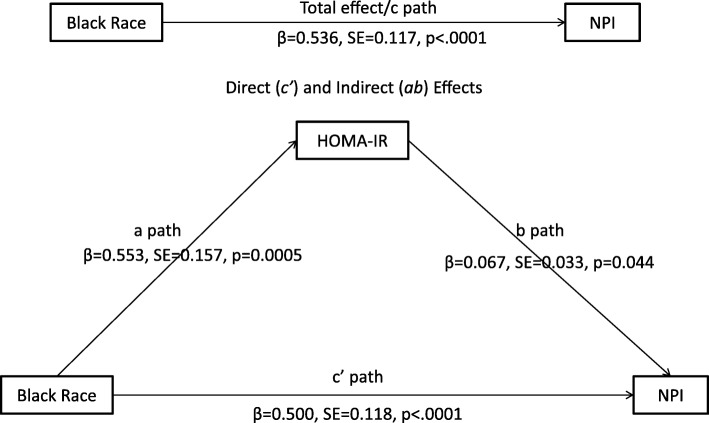
The linear mediation model showing the direct and indirect effects of race on breast cancer prognosis. The total effect (c path) of race on prognosis (NPI) and the direct (c’ path) and indirect (ab paths) are shown. HOMA-IR homeostatic model assessment of insulin resistance, NPI Nottingham Prognostic Index, *β* parameter estimate, SE standard error. Proportion of the effect that was mediated (PEM) = ab/c = 0.5532 × 0.0666/0.5364 = 7%

Insulin resistance (HOMA-IR) was positively associated with NPI scores (*r* = 0.1; *p* = 0.02). Poor prognosis was associated with younger age (55.3 ± 12.7 vs 58.9 ± 12.3 years; *p* = 0.01), and a diet that was not “very good/excellent” (58% vs 44%, *p* = 0.02), but was not associated with metabolic syndrome, smoking, drinking, or exercise. To evaluate whether HOMA-IR mediated the effect of race on NPI, the multivariate linear mediation model was used, adjusting for age. The total relationship between being a Black woman and having a worse NPI was 0.54, *p* < 0.0001; the direct effect of being a Black woman to NPI was 0.50, *p* < 0.0001; and the direct effect of HOMA-IR on NPI was 0.067, *p* = 0.04, The indirect effect of race on NPI through HOMA-IR was 0.04 (*p* = 0.002) adjusting for age (Fig. 1). This model indicates that insulin resistance (measured by HOMA-IR) significantly mediated the association between race and poor prognosis, measured by NPI score.

### Breast cancer insulin receptor and insulin-like growth factor receptor expression

**Fig. 2:**
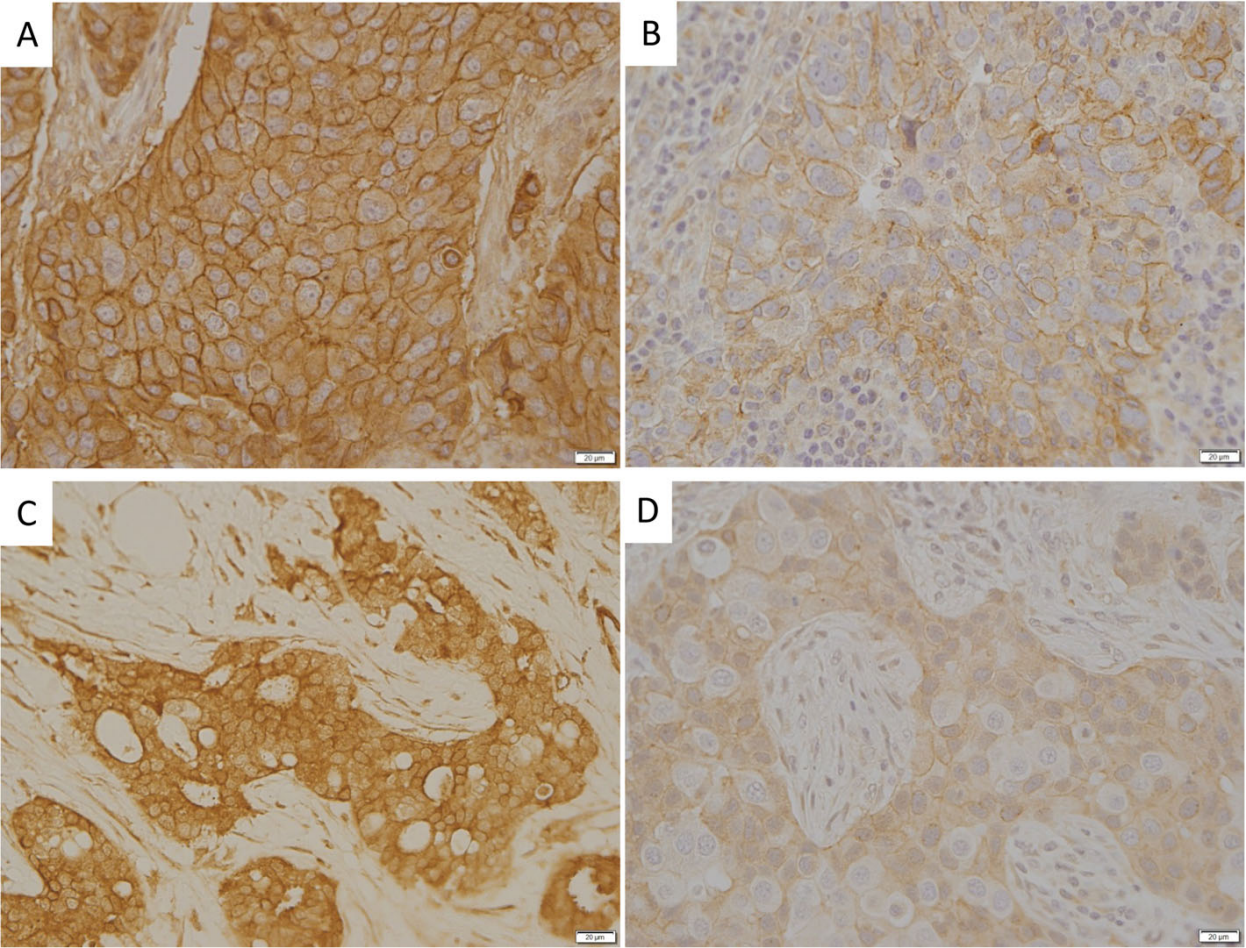
Representative images of high and low IGF-1R and IR expression by IHC. **a** High IGF-1R expression. **b** Low IGF-1R expression. **c** High IR expression. **d** Low IR expression

IR and IGF-1R IHC staining was performed on 196 available tumor specimens (Table 2). Of them, 34 tumor specimens were from Black women (17%) and 162 (83%) were from White women. One hundred sixty-eight were ER positive, 12 (6.2%) were HER2 positive by IHC, and 11 (5.7%) were TNBCs. One hundred twenty-eight (65%) specimens had high IGF-1R expression, and 68 (35%) had low IGF-1R expression. One hundred twelve (57%) of tumors exhibited high IR expression, and 84 (43%) specimens had low expression (representative images Fig. 2). More ER-positive than ER-negative tumors had high IGF-1R expression (71% vs 35%, *p* = 0.0006). These findings were consistent with previously published studies examining IGF1R RNA and protein expression in breast cancer subtypes [31–33]. No differences in IR expression were seen based on ER. TNBC exhibited no difference in intensity of IGF-1R (67% vs 45%, *p* = 0.2) or IR staining (73% vs 57%, *p* = 0.4) compared with other breast cancer subtypes, although there were only 11 cases of TNBC in the group.

**Table 2:** Patient and tumor characteristics based on IHC expression of IR, IGF-1R, and IR/IGF-1R ratio

	IR			IGF1R			IR/IGF1R			
	Low	High	***p*** value	Low	High	***p*** value	< 1	1	> 1	***p*** value
Number of cases (%)	84 (43%)	112 (57%)		68 (35%)	128 (65%)		101 (52%)	64 (33%)	30 (15%)	
Age (mean ± SD, years)	58.7 ± 11.4	58.7 ± 12.9	0.985	58.9 ± 12.4	58.6 ± 12.2	0.862	58.1 ± 12.1	60.0 ± 11.3	57.5 ± 14.7	0.530
**Race**			0.004			0.430				0.003
White	77 (48%)	85 (52%)		54 (33%)	108 (67%)		89 (55%)	54 (34%)	18 (11%)	
Black	7 (21%)	27 (79%)		14 (41%)	20 (59%)		12 (35%)	10 (29%)	12 (35%)	
**Metabolic characteristics**										
BMI (mean ± SD)	26.6 ± 5.7	28.2 ± 6.9	0.091	28.0 ± 5.7	27.2 ± 6.9	0.461	26.5 ± 6.4	27.7 ± 6.7	30.0 ± 5.7	0.039
Waist circumference (mean ± SD)	93.1 ± 15.2	100.4 ± 14.7	0.002	96.1 ± 14.5	98.0 ± 15.8	0.438	95.6 ± 15.2	99.3 ± 15.6	100.3 ± 13.2	0.203
HOMA-IR score, (mean ± SD)	1.2 ± 1.1	1.3 ± 1.1	0.635	1.3 ± 1.1	1.3 ± 1.1	0.649	1.2 ± 1.1	1.3 ± 1.0	1.6 ± 1.3	0.321
**Tumor characteristics**										
TNBC	3 (27%)	8 (73%)	0.363	6 (55%)	5 (45%)	0.191	2 (18%)	6 (55%)	3 (27%)	0.051
ER positive	69 (41%)	99 (59%)	0.391	49 (29%)	119 (71%)	< .001	96 (57%)	47 (28%)	24 (14%)	< .001
HER2 positive	4 (33%)	8 (67%)	0.565	8 (67%)	4 (33%)	0.025	3 (25%)	4 (33%)	5 (42%)	0.032
NPI (mean ± SD)	3.6 ± 0.8	3.8 ± 1.0	0.167	4.1 ± 0.8	3.6 ± 1.0	< .001	3.5 ± 1.0	3.9 ± 0.8	4.2 ± 0.8	< .001
iNPI (mean ± SD)	3.0 ± 1.1	3.2 ± 1.2	0.443	3.6 ± 1.1	2.9 ± 1.1	< .001	2.8 ± 1.2	3.3 ± 1.1	3.8 ± 1.1	< .001

There was no difference in high expression of IGF-1R by race (Black women 59% vs White women 67%, *p* = 0.38), but more Black women than White women had tumors with high IR expression (79% vs 52%, *p* = 0.004). Tumors with high IGF-1R expression had significantly better prognosis compared with tumors with low IGF-1R expression, as indicated by lower NPI scores (3.6 vs 4.1, *p* = 0.0002) and iNPI (2.9 vs 3.6, *p* < 0.0001). There was no difference in prognostic scores between IR expression categories. Women with tumors that had high IR expression had larger waist circumference than women that had tumors with low IR expression (100.4 ± 14.7 vs 93.1 ± 15.2, *p* = 0.002).

Previous studies have reported that the ratio of IR/IGF-1R expression is important in determining the sensitivity of the cancer cells to the effects of insulin [34]. We found that more Black women than White women had an IR/IGF-1R ratio > 1. Additionally, an IR/IGF-1R ratio of > 1 was associated with highest NPI (4.3) and iNPI (3.8), those with similar levels of expression IR and IGF-1R (ratio = 1) had NPI 3.9 and iNPI 3.3, and those with an IR/IGF-1R ratio < 1 had the lowest NPI (3.5) and iNPI (2.8) scores, *p* < 0.0001 for both NPI and iNPI comparisons.

## Discussion

This is the first study to examine in detail the role of insulin resistance in the disparities in breast cancer prognosis between Black women and White women. Herein, we found that insulin resistance was more prevalent in Black women with invasive breast cancer than White women, and insulin resistance mediated in part the link between race and breast cancer prognosis (determined by NPI). Additionally, we found that breast cancers from Black women had higher IR expression and were more likely to have an elevated IR/IGF-1R ratio than tumors from White women. These findings may indicate that Black women are not only more likely to have hyperinsulinemia, but are also more susceptible to the tumor promoting effects of elevated insulin by direct effects of insulin on tumor IR signaling.

We found no differences in breast cancer screening rates or in the AJCC stage at presentation between Black women and White women. Types of insurance were also similar between the two groups. However, despite these similarities, prognosis as quantified by the NPI score was worse in Black women, compared with White women. These data suggest that access to healthcare was not a major contributing factor to the differences in breast cancer prognosis in our study.

A number of lifestyle differences were noted. Black women had lower rates of smoking, alcohol consumption, exercise, and self-described healthy eating habits than White women. Significant differences in education and annual income were observed between the two groups, and Black women had more comorbidities than White women. Diet and exercise are considered to be modifiable risk factors for a number of metabolic diseases, and diet-induced weight loss has been found to improve insulin sensitivity similarly in Black and White women [35]. The differences in insulin resistance may also be related to differences in insulin metabolism between races. Previous studies have found that African American women have higher circulating insulin levels than European American women, due to reduced hepatic insulin clearance [17, 36]. Similar results have been reported in children [37]. Furthermore, some studies have reported that African American women have earlier menopause than White women, which may also contribute to a higher prevalence of metabolic syndrome in Black women [38, 39]. Based on these previous studies, and the results of our current study, it is possible that the Black women may have long-term exposure to high circulating insulin levels. These high insulin levels may contribute to the development of breast cancer subtypes that carry a poor prognosis.

Our examination of IR and IGF-1R by IHC found that high IGF-1R expression was found in ER-positive tumors and those with better prognosis by NPI. These findings are consistent with studies examining IGF1R expression at an RNA level and survival in the Molecular Taxonomy of Breast Cancer International Consortium (METABRIC) and The Cancer Genome Atlas (TCGA) datasets [31, 32]. Similarly, high IGF-1R expression by IHC has previously been associated with ER positivity in breast cancer [33, 40–43]. We found no association between IR expression and ER or HER2 status, which is also similar to previous findings [33]. Some previous studies have reported decreased survival in women with high tumor IR expression [44, 45], although not all studies have reported this association [33]. In our current study, we found no association between IR expression and prognosis; however, we did find having higher IR/IGF-1R ratio was associated with a worse prognosis. Preclinical studies have reported that higher IR/IGF-1R ratio may be indicative of cells that are more sensitive to the growth promoting effects of insulin, and resistance to therapies [34, 46]. In our current study, we found that more Black women had tumors with high IR expression and high IR/IGF-1R ratio. In the context of previously published studies, these results suggest that Black women may be more susceptible to the cancer promoting effects of hyperinsulinemia, due to higher levels of circulating insulin and higher ratio of tumor IR/IGF-1R expression.

Limitations include the cross-sectional design, which meant we were unable to determine the duration of insulin resistance prior to breast cancer diagnosis and led us to determine prognosis through the NPI. However, the NPI has previously been well-validated as a prognostic indicator in different populations [47]. Additionally, it predicts prognosis independent of any future differences in breast cancer treatments that may contribute to disparities in breast cancer survival. We also had incomplete data on some breast cancer risk factors, including reproductive history and parity. The prevalence of obesity as defined by BMI in our study was lower than anticipated for a US population. Recent US population studies report that 35.5% of White women and 56.9% of Black women ages 20 years and over are obese [48]. The lower prevalence of obesity in our study is likely related to the geographical regions in which this study was performed. The prevalence of self-reported obesity by BMI among US adults in New York, New Jersey, and Connecticut are between 25 and 30% [49]. Our relatively low prevalence of obesity is also likely to explain the lower than anticipated HOMA-IR results. The NHANES III reported that 25% of US adults without diabetes have HOMA-IR scores > 2.8 [21]. In our population, only 11% of White women and 17% of Black women had HOMA-IR scores of > 2.8. While the focus of our study was to examine the link between insulin resistance and disparities in prognosis in women with newly diagnosed breast cancer, we acknowledge that this is certainly just one of a number of complex factors that contribute to racial disparities in breast cancer prognosis.

## Conclusions

Overall, our study finds that insulin resistance is one factor that contributes to the worse prognosis in breast cancer between Black and White women, potentially through direct effects of insulin on the tumor IR. Given the differences in circulating insulin levels and tumor IR expression between Black women and White women, it will be important in future studies to explore whether lowering insulin levels or targeting IR signaling will improve breast cancer survival disparities.

## Supplementary information

**Additional file 1.** Supplementary Methods.

## Data Availability

The data that support the findings in this study are not publically available; however, data are available from the authors upon reasonable request and with permission of the participating sites’ institutional review boards.

## Abbreviations

ER: Estrogen receptor
HER2: Human epidermal growth factor receptor 2
HOMA-IR: Homeostatic model assessment of insulin resistance
IGF-1R: Insulin-like growth factor receptor
IR: Insulin receptor
MetS: Metabolic syndrome
NPI: Nottingham Prognostic Index
PR: Progesterone receptor
TNBC: Triple-negative breast cancer
