# Supplementary material for: Insulin resistance contributes to racial disparities in breast cancer prognosis in US women
Source: Breast Cancer Res. 2020 May 12;22:40. doi: 10.1186/s13058-020-01281-y (PMC7216707; doi:10.1186/s13058-020-01281-y)
Supplement: Supplementary file 1 — Additional file 1. Supplementary Methods. [file 13058_2020_1281_MOESM1_ESM.docx]

Supplementary Methods.

Immunohistochemistry Staining:

Formalin-fixed, paraffin-embedded breast cancer tissues were obtained through the pathology / histology departments of each institution. 5μm sections were mounted on slides, de-identified, and stained manually by IHC. Prior to staining, slides were stored at 4^0^C. Antigen retrieval was performed using microwave heating in sodium citrate buffer (pH 6) for IR staining, and tris EDTA buffer (pH 9) for IGF-1R staining. Antibodies used were: IRβ (ab137747, Abcam, Eugene, OR) 1:300 dilution, and IGF-1Rβ (#3027, Cell Signaling Technologies, Carlsbad, CA) 1:400 dilution. IR and IGF-1R detection and staining were performed using the avidin-biotin (Vector Laboratories, Burlingame, CA) and polymer-based detection (Cell Signaling Technologies) methods, respectively. Images were obtained using the Olympus AX70 microscope and quantified using the CellSens analysis software (Olympus, Center Valley, PA) by investigators blinded to the patients’ clinical data.
